# Evaluation of Antibiotic Prescribing Practices and Antimicrobial Sensitivity Patterns in Urinary Tract Related Infectious Diseases in Pediatric Patients

**DOI:** 10.3389/fped.2021.740106

**Published:** 2021-12-23

**Authors:** Sirajudeen S. Alavudeen, Anas Ali Asiri, Shatha Abdulrahman Fageeh, Ahmed Abdoh Aljarie, Mir Javid Iqbal, Noohu Abdulla Khan, Fauzia Tabassum, Mohamed Rahamathulla, Umme Hani, Md Sayeed Akhtar

**Affiliations:** ^1^Department of Clinical Pharmacy, College of Pharmacy, King Khalid University, Abha, Saudi Arabia; ^2^Department of Paediatrics, Pediatric Infectious Diseases, Abha Maternity and Children Hospital, Abha, Saudi Arabia; ^3^Department of Pharmaceutical Sciences, Northeastern University, Boston, MA, United States; ^4^Department of Pharmacology, Santosh Medical College and Hospital, Ghaziabad, India; ^5^Department of Pharmaceutics, College of Pharmacy, King Khalid University, Abha, Saudi Arabia

**Keywords:** urinary tract infections, pediatric, *Escherichia coli*, sensitivity pattern, antibacterial resistance

## Abstract

Complications of urinary tract infections (UTIs) like kidney failure and septicaemia develop once infections spread from the upper urinary tract to other parts of the body by haematogenous dissemination and they pose great health and economic burden to the countries. This retrospective study was conducted among 132 patients with bacterial UTIs in the inpatient department of tertiary care hospital in Abha, Saudi Arabia. During the study period, *Escherichia coli* (*E. coli*) and *Klebsiella pneumonia* (*K. pneumonia*) along with other 15 different bacteria were isolated. A significant difference (*P* < 0.05) was observed between the male and female children population in different age groups. We observed fever (84.09%) as a major symptom (*P* < 0.05), and seizure (9%) was reported as a major concomitant condition among UTI cases. Around 31.82% of *E. coli* was found to be the most common uropathogens in pediatric cases followed by 25% in *K. pneumoniae*. *E. coli* was observed to be more susceptible (92.86%) to amikacin, ceftriaxone, levofloxacin, ertapenem, gentamycin, meropenem, piperacillin-tazobactam, tigecycline, and ceftazidime. However, meropenem, tigecycline, and amikacin were observed to be effective in 100% of cases of *K. pneumoniae*. Meanwhile, cephalosporins were the most commonly prescribed drug category among different classes of drugs. Almost 99% of pediatric cases, based on their age, were admitted to the ward, and drugs were administered intravenously. We concluded that microbiology laboratory evidence on the causative organisms and choice of treatment together allows tailoring appropriate treatment regimens in conjunction with clinical experiences.

## Introduction

Urinary tract infection (UTI) is defined by the presence of more than 10^5^ colony-forming unit per milliliter (CFU/ml) of urine with a multiplication of organisms in the urinary tract (1). Worldwide, Centers for Disease Control and Prevention (CDC) 2017 in National Health prevalence survey reported UTI prevalence rate of 0.48%, and among them, 80% were symptomatic UTI cases. It poses a great economic burden to the countries (2, 3). Complications of UTIs mainly include kidney failure due to extensive renal damage and sepsis, which occurs when the infection spreads from the lower urinary tract to other parts of the body. UTIs, particularly uncomplicated UTIs like acute uncomplicated cystitis and acute uncomplicated pyelonephritis, are the most frequent reasons for consulting a general practitioner in most of the developed countries, which are being treated by antibiotics (4, 5). The contribution of primary healthcare is particularly important, as this is where almost 80% of all antibiotics used within the health service are prescribed (6). Antimicrobial resistance is an internationally recognized threat to health. The resistance to antibiotics in bacterial infections limits the availability of effective treatment options, rendering some commonly encountered bacterial infections difficult to treat, including UTIs (7, 8). Antibiotic-resistant infections are also twice as likely to be associated with greater morbidity and mortality concurrent to increased healthcare costs (9). Sensitivity analysis determines the effectiveness of antibiotics against microorganisms such as bacteria that have been isolated from cultures. In the future, the rate of antibiotic resistance will possibly continue to increase, and thus, building policies to use the antibiotics to decrease this trend is the need of the hour and must be incorporated in urological practice. Antibiotic sensitivity pattern is an important tool to choose the right antibiotics by the clinicians for the patient with UTIs (10, 11). Children receive a lot of primary healthcare services and receive a disproportionately higher number of antibiotics compared with middle-aged populations (12). It is also found that the prevalence of resistance to commonly prescribed antibiotics in primary care among children with UTIs due to *Escherichia coli* (*E. coli*) is high, particularly in countries outside the Organization for Economic Co-operation and Development, where the antibiotics are available over the counter (13). Based on the above points, it was considered suitable by the investigator to study the drug utilization and antibiotic sensitivity pattern of bacterial pathogens in patients suffering from UTI. Moreover, this UTI-related study will help in establishing a proper antibiotic utilization guideline as well as in promoting the rational prescribing of antibiotics. Hence, keeping these facts in the mind, this study was conducted to assess the drug of choice for the empirical antibiotic therapy and to find the changing susceptibility pattern of urinary pathogens to commonly used antimicrobials among urinary tract-related infectious diseases.

## Materials and Methods

A retrospective study was conducted to study and assess the urinary tract-related bacterial infections in the inpatient department of a tertiary care teaching hospital in Abha, Saudi Arabia. The data was collected between October 2020 and March 2021. The sample size of the study was estimated by assuming α-error of 0.05, power of 80%, and percentage of cases having UTIs to be 5% based on previous studies. Total 250 records were reviewed, and 132 subjects were taken into consideration for this study as per study criteria. A urine sample was collected by both urinary catheterization and mid-stream urine (MSU) method as per the standard procedure. Positive urine cultures were designated as those with ≥10^5^ CFU/ml of a single identified bacterial species. Cultures with colonies <10^5^ CFU/ml and those already on antibiotic drugs were excluded from the study. Relevant data related to microbiological isolates from confirmed cases of UTIs under the age of 10 years were included in this study. Data also excluded isolated organisms other than bacteria such as candida, incomplete antibiotic prescriptions, patients with acute complicated UTIs (e.g., acute pyelonephritis and UTIs with sepsis or bacteremia), catheter-associated UTI, and major comorbidities (such as liver disease, renal insufficiency, malignant tumor, and AIDS) from this study. The data of selected patients were recorded in drug utilization and microbiological sensitivity documentation form from physician prescribing records and the microbiology laboratory data. The study protocol [(ECM#2020-241)–(HAPO-06-B-001)] was reviewed and approved by the Institutional Ethics Committee of King Khalid University, Guraiger, Abha, Saudi Arabia.

All statistical analysis was performed by using SPSS software, version 21 (SPSS, Chicago, IL, USA). Based on the type of data, an appropriate statistical test like *t*-test, analysis of variance (ANOVA), and chi-square test were applied to analyze the data. Descriptive statistics were summarized by using frequency and percentage (%). A *P* value <0.05 was considered statistically significant.

## Results

A total of 132 patients, having confirmed UTIs were included in this study. During the study period, *E. coli* and *K. pneumonia* along with other 15 different bacteria were isolated.

### Age and Gender

The majority of patients (43.18%) were in the age group between 2 and 12 months, and the number of male patients was more in this age group (27.27%). A significant difference (*P* < 0.05) was observed between the male and female children population in different age groups. Among the 132 patients, 25 (56.82%) were males and 19 (43.18%) were females, indicating UTI is more prevalent in the male in comparison to females in the lower age group, and a statistically significant difference (*P* < 0.05) was observed among them (Table 1).

**Table 1:** Distribution of urinary tract infection cases according to age.

**Gender**	**Age group**				***P*-value**
	**0–2**	**>2–12**	**>12–48**	**>48**	
Male	24 (18.18)	36 (27.27)	9 (27.27)	6 (27.27)	0.001
*N* = 25 (56.82%)					
Female	12 (9.09)	21 (15.91)	6 (4.55)	18 (13.64)	
*N* = 19 (43.18%)					
Total (%)	36 (13.64)	57 (43.18)	15 (11.36)	24 (18.18)	

*N and % represents number and percentage respectively. P value < 0.05 was considered as statistically significant difference*.

### Drug and Food Allergy

We observed that 3.78% of total children reported allergies to either milk or fish. However, no drug allergy was reported as patient history or during the treatment.

### Patient's Medical History and Symptoms at the Time of Hospitalization

As mentioned in Table 2A, we observed fever (84.09%) as a major symptom (*P* < 0.05) of UTIs followed by diarrhea (18.18%) and vomiting (6%). Concurrently, seizure (9%) was observed as a concomitant condition in children suffering from UTIs. Others include, developmental dysplasia, end stage renal disease and congenital heart diseases mentioned in Table 2B.

**Table 2:** Presence of symptoms, concomitant conditions, and pathogen distributions among children with urinary tract infections.

	***N***	**%**	***P-*value**
**(A) TYPE OF SYMPTOMS**			
Fever	111	84.09*	*P* < 0.004
Diarrhea	24	18.18	
Cough	6	4.55	
Vesicoureteral reflux	3	2.27	
Chronic diarrhea	3	2.27	
Vomiting	9	6.82	
Crying during micturition	3	2.27	
Bronchiolitis	6	4.55	
**(B) CONCOMITANT CONDITION**			
Seizure	12	9.09	*P* > 0.05
Developmental dysplasia of the hip	6	4.54	
End stage renal disease	3	2.27	
Coronary heart disease	3	2.27	
Hypertension, hydronephrosis	3	2.27	
Post cardiac arrest, Covid-19	3	2.27	
Hypothyroidism	3	2.27	
Broncho pneumonia	3	2.27	
Convulsion-Dany walker syndrome-glaucoma	3	2.27	
Pneumonia	3	2.27	
Bladder mass	3	2.27	
Cyanosis	3	2.27	
Bilateral hydronephrosis	3	2.27	
Hydronephrosis	3	2.27	
Hydrocephalus	3	2.27	
Microcytic anemia	3	2.27	
None	72	54.54545	
**(C) DISTRIBUTION OF UROPATHOGENS CAUSING URINARY TRACT**			
**INFECTION**			
**Name of pathogens**			
*Escherichia coli* (*E. coli*)	42	31.82	*P* > 0.05
*Klebsiella pneumoniae*	33	25.00	
*Pseudomonas aeruginosa*	9	6.82	
*Enterococcus faecium*	9	6.82	
*Klebsiella oxytoca*	6	4.55	
*Mixed infections*	6	4.55	
*Klebsiella pneumonia* *and Pseudomonas aeruginosa*	3	2.27	
*Klebsiella pneumoniae and E. coli*	3	2.27	
*Staphylococcus aureus*	3	2.27	
*Enterobacter colacae*	3	2.27	
*Acinetobacter MDR*	3	2.27	
*Staphylococcus epidermidis*	3	2.27	
*Septicemia* (*Multiple infection*)	3	2.27	
*Actinetobacterbaumannii complex*	3	2.27	
*Stenotrophomonas maltophilia*	3	2.27	

*N and % represents number and percentage respectively. P value < 0.05 was considered as statistically significant difference*.

### Presence of Uropathogens Causing Urinary Tract Infection

As mentioned in Table 2C, *E. coli* was found to be the most common uropathogen (31.82%) in pediatric cases followed by *K. pneumoniae* (25%), *Pseudomonas aeruginosa* (*P. aeruginosa*; 6.82%), and *Enterococcus faecium* (*E. faecium*; 6.82%). Other microbiological isolates included *Klebsiella oxytoca* (*K. oxytoca*), *Staphylococcus aureus* (*S. aureus*), *Enterobacter cloacae, Acinetobacter MDR* (*A. MDR*), *Staphylococcus epidermidis* (*S. epidermis*), *Actinetobacter baumannii complex* (ABC), *Stenotrophomonas maltophilia* (*S. maltophilia*), and septicemia (multiple infections).

### Sensitivity Pattern

*E. coli* was observed to be more susceptible to amikacin (92.86%), ceftriaxone (92.86%), levofloxacin (92.86%), ertapenem (92.86%), gentamycin (92.86%), meropenem (92.86%), piperacillin-tazobactam (92.86%), tigecycline (92.86%), and ceftazidime (92.86%) followed by cefepime (78.57%), imipenem (78.57%), co-trimoxazole (78.57%), cefotaxime (71.43%), cefoxitin (71.43%), and ciprofloxacin (71.43%). However, meropenem, tigecycline, and amikacin were observed to be effective in 100%, 81.82%, and 72.73% cases of *K. pneumonia*, respectively followed by co-trimoxazole (63.64%), ertapenem (63.67%), cefotaxime (54.44%), and imipenem (54.55%). In case of *P. aeruginosa*, 100% of cases were susceptible to cefepime, ciprofloxacin, levofloxacin, gentamycin, ceftazidime, and piperacillin-tazobactam. However, amikacin, imipenem, meropenem, and aztreonam were observed to be effective in 66.67% of cases. *E. faecium* was observed to be susceptible with tigecycline, linezolid, and vancomycin in 100% of cases followed by quinupristin/dalfopristin (66.67%) and teicoplanin (66.67%). Other cases related to *K. oxytoca, S. aureus, E. cloacae, A. MDR, S. epidermidis, ABC*, and *S. maltophilia* and its sensitivity to antimicrobial agents are mentioned in Table 3.

**Table 3:** Antibiotic sensitivity pattern of common uropathogens in urinary tract infections.

**Antibiotics**	***E. coli* (*N* = 42)**	**%**	***K. pneumonia* (*N* = 33)**	**%**	***P. aeruginosa* (*N* = 9)**	**%**	***E. faecium* (*N* = 3)**	**%**	***K. oxytoca* (*N* = 2)**	**%**
Amikacin	39	92.86	*24*	72.73	6	66.67	–		6	100
AM-CL	21	50.00	9	27.27	–	–	–		–	–
Ampicillin	0	00.00	0	0.00	–	–	3	33.33	–	–
Cefepime	33	78.57	6	18.18	9	100.00	–		6	100
Cefoxitin	30	71.43	15	45.45	–	–	–		–	–
Ceftriaxone	39	92.86	6	18.18	–	–	–		–	–
Cefuroxime	18	42.86	6	18.18	–	–	–		–	–
Ciprofloxacin	30	71.43	12	36.36	9	100.00	–		6	100
Levofloxacin	39	92.86	18	54.55	9	100.00	3	33.33	6	100
Ertapenem	39	92.86	21	63.64	–	–	–		6	100
Gentamicin	39	92.86	15	45.45	9	100.00	3	33.33	6	100
Imipenem	33	78.57	18	54.55	6	66.67	–		–	–
Meropenem	39	92.86	33	100.00	6	66.67	–		6	100
Nitrofurantoin	18	42.86	15	45.45	–	–	3	33.33	–	–
Piperacillin-Tazobactam	39	92.86	15	45.45	9	100.00	–		6	100
Tigecycline	39	92.86	27	81.82	–	–	9	100.00	6	100
Co-trimoxazole	33	78.57	21	63.64	–	–	–		6	100
Cefotaxime	30	71.43	18	54.55	–	–	3	33.33	–	
Ceftazidime	39	92.86	6	18.18	9	100.00	–		6	100
Fosfomycin	6	14.29	12	36.36	–	–	–		–	–
Quinupristin/ Dalfopristin	–	–	–	–	–	–	6	66.67	–	–
Linezolid	–	–	–	–	–	–	9	100.00	–	–
Vancomycin	–	–	–	–	–	–	9	100.00	–	–
Teicoplanin	–	–	–	–	–	–	6	66.67	–	–
Streptomycin (High dose)	–	–	–	–	–	–	3	33.33	–	–
Aztreonam	–	–	–	–	6	66.67	–		6	100
Colistin	–	–	–	–	3	33.33	–		–	–
Clindamycin	–	–	–	–	–	–	–		–	–
Daptomycin	–	–	–	–	–	–	–		–	–
Tobramycin	–	–	–	–	–	–	–		6	100
**Antibiotic sensitivity pattern of other different uropathogens in urinary tract infections**										
Staphylococcus aureus	Ciprofloxacin, Clindamycin, Daptomycin, Erythromycin, Fosfomycin, Linezolid, Moxifloxacin, Mupirocin, Rifampin, Synercid, Teicoplanin, Tetracycline, and Vancomycin									
Enterobacter cloacae	Amikacin, Ciprofloxacin, Gentamicin, Levofloxacin, Meropenem, Norfloxacin, and Tobramycin									
Acinetobacter MDR*	Vancomycin, Ceftazidime, and Rifampin									
Staphylococcus epidermidis	Vancomycin, Tetracycline, Nitrofurantoin, Rifampicin, and Bactrim									
Acinetobacter baumannii complex	Colistin-tigecycline									
Stenotrophomonas maltophilia	Ceftazidime, Levofloxacin, and Co-trimoxazole									

*N and % represents number and percentage respectively about sensitivity for each isolated organism. AM-CL, Amoxicillin-Clavulanic acid; E. coli, Escherichia coli; K. pneumoniae, Klebsiella pneumoniae; P. aeruginosa, Pseudomonas aeruginosa; E. faecium, Enterococcus faecium; K. oxytoca, Klebsiella oxytoca*.

### Utilization Pattern of Antibiotics

Ceftriaxone (29.55%) was the most commonly prescribed drug in UTIs followed by vancomycin (25%), meropenem (20.45%), and cefuroxime (15.91%). Meanwhile, cephalosporins were most commonly prescribed drug category among different classes of drugs (Table 4).

**Table 4:** Usage of antibiotics in different cases of urinary tract infections.

**Class of drugs**	**Drugs**	***N***	**%**
Penicillin	Amikacin	6	4.55
	Gentamicin	3	2.27
	Amoxicillin/	3	2.27
	Potassium Clavulanate		
	Ampicillin	15	11.36
Cephalosporins	Ceftriaxone	39	29.55
	Cefuroxime	21	15.91
	Cefotaxime	18	13.64
	Ceftazidime	9	6.82
Fluroquinolones	Ciprofloxacin	3	2.27
	Levofloxacin	3	2.27
Tetracycline	Tigecycline	3	2.27
Sulphonamides	Co-trimoxazole	12	9.09
Polymyxins	Colistin	3	2.27
Macrolides	Clindamycin	9	6.82
	Azithromycin	6	4.55
Anti-mycobacterial	Rifampin	9	6.82
	Meropenem	27	20.45
Others	Nitrofurantoin	3	2.27
	Linezolid	6	4.55
	Vancomycin	33	25

*N and % represents number and percentage respectively about usage of antibiotics in different cases of urinary tract infections*.

### Dosage Form

Almost 99% of pediatric cases, based on their age, were admitted to the ward, and drugs were administered intravenously.

### Dose Calculation

The dose of the prescribed drug was calculated based on body weight (BW) as per recommended dose in mg/kg of BW.

## Discussion

The goal of this study was to evaluate the antibiotic sensitivity pattern of microbiological isolates and antibiotic recommendation in pediatric cases suffering from UTIs admitted in the pediatric ward of a tertiary hospital, Abha, Saudi Arabia. Antimicrobial susceptibility testing of individual isolates is important to confirm sensitivity to chosen empirical antimicrobial agents. Prevalence was highly influenced by the gender and age of the patients. The present study demonstrated a higher incidence of infections in males in comparison to female patients in contrast to previous studies (7). Digging deeper, we observed that male to female proportion of UTIs cases was much greater in between the age of 0–2 months (2:1), and this proportion almost reverses in higher age group children (1:3). The most chances of higher prevalence in male neonates and infants are due to poor hygiene particularly due to lack of circumcision as reported in earlier studies (14). However, the UTIs were reported to be more prevalent in the female young and middle-aged population and thought to be due to lesser distance between the anus (the usual source of uropathogens) and the urethral meatus, the greater length of the male urethra, and the antibacterial activity of prostatic fluid in male (15). Furthermore, this may be due to the vaginal colonization with uropathogens or entry of colonizing uropathogens into the bladder *via* urethra (5, 16). This is well-evidenced that many drugs, as well as foods like milk, egg nuts, and seafood, can trigger anaphylactic reactions in children (17, 18). Concurrent to this, we also observed an allergy to fish and milk in our study. But no drug allergy was noticed in any patients indicating the safety of drugs prescribed among the patients. Pyelonephritis has been reported to be a major cause of pain and fever in UTIs (19). Consistent with previous study, we observed severe fever, diarrhea, and vomiting as the commonest symptoms for hospital admission (20). Moving forward, we also observed seizure as the most common concomitant condition in children having UTIs. In line of the above discussion, fever was a major symptom in UTIs and this febrile seizure in pediatric population may be due to raised body temperature (21). *E. coli* was the most causative organism of UTIs and has been reported to be present in 75–90% of UTI isolates (22). Concurrently, the present study confirmed a higher prevalence of isolates of *E. coli* (31.82%) followed by *K. pneumoniae* (25%). Moreover, many more microorganisms like *P. aeruginosa* (6.82%), *E. faecium* (6.82%), and *K. oxytoca* (4.55%) have been also reported to be prevalent in our study. These findings indicated a prevalence of the highly diverse type of microbes in UTIs as reported earlier (23, 24). The present study showed that *E. coli* have sensitivity in 92.86% of cases, to amikacin, ceftriaxone, levofloxacin, ertapenem, gentamycin, meropenem, piperacillin-tazobactam, tigecycline, and ceftazidime (25). Among antibiotics, ampicillin showed high resistance in the case of *E. coli* isolates (100%) like previous studies (7, 26, 27). Therefore, rational use of antibiotics must be implemented after keeping these facts in mind. Concurrently, meropenem was also observed to be effective in 100% of cases of *K. pneumonia* followed by tigecycline and amikacin as reported earlier (28, 29). As mentioned above, various other isolates were also reported in our study. In case of *P. aeruginosa*, 100% of cases were susceptible to cefepime, ciprofloxacin, levofloxacin, gentamycin, ceftazidime, and piperacillin-tazobactam (30). However, amikacin, imipenem, meropenem, and aztreonam were effective in 66.67% of cases (31). Studies reported that among *enterococci, E. faecalis* play a major role in various infections. However, we observed *E. faeciumas* UTI isolates and reported to be susceptible with tigecycline, linezolid, and vancomycin in 100% of cases followed by quinupristin/dalfopristin and teicoplanin (32, 33). Other cases related to *K. oxytoca, S. aureus, E. cloacae, A. MDR, S. epidermidis, ABC*, and *S. maltophilia* and its sensitivity to antimicrobial agents were in line with previous studies (23, 24). Looking forward, ceftriaxone (29.55%), vancomycin (25%), meropenem (20.45%), and cefuroxime (15.91%) have been reported to be the four most commonly prescribed antibiotics in UTIs in children. The reason behind this may be ease of route of drug administration and bacterial sensitivity to antibiotics in pediatric population. Almost all drugs were administered intravenously possibly due to the nature of antibiotics and treatment recommendations about the route of drug administration in children. Various authors reported similar evidence in different studies (22, 34, 35). Our study highlights the continuous implementation of rational use of antibiotics in case of UTIs. Also, we strongly recommend an antibiotic sensitivity test before deciding treatment plan for UTIs considering proper disease management and its prevention toward multidrug resistance (36). This is the only way to keep the treatment plan on track and should be carried out frequently to keep eye on resistance to antibiotics in the different clinical setups.

## Conclusions

The emergence of multidrug-resistant organisms poses a great threat to clinicians in the prevention and management of UTIs and their related complications. This real-life data has vital importance in rationalizing the use of antibiotics to prevent the emergence of drug-resistant cases. Reasonable evidence on the causative organisms and choice of treatment together allows deciding appropriate treatment plans made in conjunction with clinical and practical considerations. Empirical use of antibiotics should be evaluated considering the drug resistance patterns, and drug formularies should be updated to maximize therapeutic benefits and development of resistant strains. The access of data to a certain extent and no differentiation between upper UTIs and lower UTIs are the major limitations of this study.

## Data Availability Statement

The datasets generated during and/or analyzed during the current study are not publicly available due to patent information confidentiality clause, but are available from the corresponding author on reasonable request. Requests to access the datasets should be directed to Sirajudeen S. Alavudeen, sshaik@kku.edu.sa.

## Ethics Statement

The study protocol [(ECM#2020-241)-(HAPO-06-B-001)] was reviewed and approved by Institutional Ethics Committee of King Khalid University, Guraiger, Abha (KSA).

## Author Contributions

All authors listed have made a substantial, direct, and intellectual contribution to the work and approved it for publication.

## Funding

This work was supported by the Dean of Scientific Research, King Khalid University, Saudi Arabia and this financial support was greatly appreciated for the group research Project under grant number (grant numbers-RGP-2, 168-42).

## Conflict of Interest

The authors declare that the research was conducted in the absence of any commercial or financial relationships that could be construed as a potential conflict of interest.

## Publisher's Note

All claims expressed in this article are solely those of the authors and do not necessarily represent those of their affiliated organizations, or those of the publisher, the editors and the reviewers. Any product that may be evaluated in this article, or claim that may be made by its manufacturer, is not guaranteed or endorsed by the publisher.

## References

[1] Davidson AM Cumming AD Swainson CP. Disease of kidney and genitourinary system. In: Edward CRW, Brucheier IAD, Hustel C, Ahifrus ER, editors. Davidson's Principle and Practice of Medicine, 17th Edn. New York, NY: Churchill Livingstone (1995). p. 611–68.

[2] Centers for Disease Control Prevention. UTI Prevalence Survey. (2019). Available online at: https://www.cdc.gov/nhsn/pdfs/training/2019/ltcf/uti-prevalence-survey-508.pdf (accesssed July 01, 2021).

[3] Nicolle LE Bradley S Colgan R Rice JC Schaeffer A Hooton TM. Infectious Diseases Society of America guidelines for the diagnosis and treatment of asymptomatic bacteriuria in adults. Clin Infect Dis. (2005) 40:643–54. 10.1086/427507 15714408

[4] Hooton TM Besser R Foxman B Fritsche TR Nicolle LE. Acute uncomplicated cystitis in an era of increasing antibiotic resistance: a proposed approach to empirical therapy. Clin Infect Dis. (2004) 39:75–80. 10.1086/422145 15206056

[5] Medina M Castillo-Pino E. An introduction to the epidemiology and burden of urinary tract infections. Ther Adv Urol. (2019) 11:1756287219832172. 10.1177/1756287219832172 31105774 PMC6502976

[6] Majeed A Moser K. Age- and sex-specific antibiotic prescribing patterns in general practice in England and Wales in (1996). Br J Gen Pract. (1999) 49:735–6. 10756619 PMC1313505

[7] Rizwan M Akhtar M Najmi AK Singh K. *Escherichia coli* and *Klebsiella pneumoniae* sensitivity/resistance pattern towards antimicrobial agents in primary and simple urinary tract infection in patients visiting university hospital of Jamia Hamdard New Delhi. Drug Res (Stuttg). (2018) 68:415–420. 10.1055/a-0576-0079 29529677

[8] Alavudeen SS Vigneshwaran E Asiri SAA Alahmari MHA Mohammed MA Algahtani T. Distribution of multi-resistant bacterial isolates from clinical specimens in a hospital environment of kingdom of Saudi Arabia. J Young Pharma. (2017) 9:347–51. 10.5530/jyp.2017.9.69

[9] Holmberg SD Solomon SL Blake PA. Health and economic impacts of antimicrobial resistance. Rev Infect Dis. (1987) 9:1065–78. 10.1093/clinids/9.6.1065 3321356

[10] Ananthanarayan R Paniker CKJ (editors). Laboratory control of antimicrobial therapy. In: Textbook of Microbiology, 6th Edn. Hyderabad: Orient Longman private Limited (2000). p. 581–3.

[11] Alakhali KM Alzomor A Alavudeen S Khan N Dawbaa S. Bacterial resistance of antibiotics used in urinary tract infection. Asian J Pharm Clin Res. (2013) 6:87–91.

[12] Ready D Lancaster H Qureshi F Bedi R Mullany P Wilson M. Effect of amoxicillin use on oral microbiota in young children. Antimicrob Agents Chemother. (2004) 48:2883–7. 10.1128/AAC.48.8.2883-2887.2004 15273096 PMC478491

[13] Bryce A Hay AD Lane IF Thornton HV Wootton M Costelloe C. Global prevalence of antibiotic resistance in paediatric urinary tract infections caused by *Escherichia coli* and association with routine use of antibiotics in primary care: systematic review and meta-analysis. BMJ. (2016) 52:i939. 10.1136/bmj.i939 26980184 PMC4793155

[14] Simoes e Silva AC Oliveira EA. Update on the approach of urinary tract infection in childhood. J Pediatr (Rio J). (2015) 91:S2–S10. 10.1016/j.jped.2015.05.003 26361319

[15] Hickling DR Sun TT Wu XR. Anatomy and physiology of the urinary tract: relation to host defense and microbial infection. Microbiol Spectr. (2015) 3:1–29. 10.1128/microbiolspec.UTI-0016-2012 26350322 PMC4566164

[16] Storme O Tirán Saucedo J Garcia-Mora A Dehesa-Davila M Naber KG. Risk factors and predisposing conditions for urinary tract infection. Ther Adv Urol. (2019) 11:1756287218814382. 10.1177/1756287218814382 31105772 PMC6502981

[17] Samady W Trainor J Smith B Gupta R. Food-induced anaphylaxis in infants and children. Ann Allergy Asthma Immunol. (2018) 121:360–5. 10.1016/j.anai.2018.05.025 29860051

[18] Seth D Poowutikul P Pansare M Kamat D. Food allergy: a review. Pediatr Ann. (2020) 49:e50–e8. 10.3928/19382359-20191206-01 31930423

[19] Belyayeva M Jeong JM. Acute pyelonephritis. In: StatPearls. Treasure Island, FL: StatPearls Publishing (2021). Available online at: https://www.ncbi.nlm.nih.gov/books/NBK519537/ (accessed July 10, 2020).

[20] Fallahzadeh MH Ghane F. Urinary tract infection in infants and children with diarrhoea. East Mediterr Health J. (2006) 12:690–4. 17333811

[21] Mahyar A Ayazi P Azimi E Dalirani R Barikani A Esmaeily S. The relation between urinary tract infection and febrile seizure. Iran J Child Neurol. (2018) 12:120–6. 30279715 PMC6160625

[22] Leung AKC Wong AHC Leung AAM Hon KL. Urinary tract infection in children. Recent Pat Inflamm Allergy Drug Discov. (2019) 131:2–18. 10.2174/1872213X13666181228154940 30592257 PMC6751349

[23] Flores-Mireles AL Walker JN Caparon M Hultgren SJ. Urinary tract infections: epidemiology, mechanisms of infection and treatment options. Nat Rev Microbiol. (2015) 13:269–84. 10.1038/nrmicro3432 25853778 PMC4457377

[24] Sopena-Sutil R Medina-Polo J Justo-Quintas J Gil-Moradillo J Garcia-Gonzalez L Benítez-Sala R. Healthcare-associated infections after lower urinary tract endoscopic surgery: analysis of risk factors, associated microorganisms and patterns of antibiotic resistance. Urol Int. (2018) 100:440–4. 10.1159/000488251 29649830

[25] Sedighi I Solgi A Amanati A Alikhani MY. Choosing the correct empirical antibiotic for urinary tract infection in pediatric: surveillance of antimicrobial susceptibility pattern of *Escherichia coli* by E-Test method. Iran J Microbiol. (2014) 6:387–91. 25926955 PMC4411423

[26] Shigemura K Kitagawa K Osawa K Yamamichi F Tokimatsu I Nomi M. Comparison of antibiotics use, urinary tract infection (UTI)-causative bacteria and their antibiotic susceptibilities among 4 hospitals with different backgrounds and regions in Japan. J Chemother. (2018) 30:31–6. 10.1080/1120009X.2017.1376817 28956738

[27] Alanazi MQ Alqahtani FY Aleanizy FS. An evaluation of *E. coli* in urinary tract infection in emergency department at KAMC in Riyadh, Saudi Arabia: retrospective study. Ann Clin MicrobiolAntimicrob. (2018) 17:3. 10.1186/s12941-018-0255-z 29422058 PMC5806437

[28] Yasin F Assad S Talpur AS Zahid M Malik SA. Combination therapy for multidrug-resistant *Klebsiella pneumoniae* urinary tract infection. Cureus. (2017) 22:9:e1503. 10.7759/cureus.1503 28948123 PMC5608481

[29] Bassetti M Righi E Carnelutti A Graziano E Russo A. Multidrug-resistant *Klebsiella pneumoniae*: challenges for treatment, prevention and infection control. Expert Rev Anti Infect Ther. (2018) 16:749–61. 10.1080/14787210.2018.1522249 30207815

[30] Narten M Rosin N Schobert M Tielen P. Susceptibility of *Pseudomonas aeruginosa* urinary tract isolates and influence of urinary tract conditions on antibiotic tolerance. Curr Microbiol. (2012) 64:7–16. 10.1007/s00284-011-0026-y 21984270

[31] Yayan J Ghebremedhin B Rasche K. Antibiotic resistance of *Pseudomonas aeruginosa* in pneumonia at a single university hospital center in Germany over a 10-year period. PLoS One. (2015) 10:e0139836. 10.1371/journal.pone.0139836 26430738 PMC4592231

[32] Rudy M Nowakowska M Wiechuła B Zientara M Radosz-Komoniewska H. Analizalekowrazliwości *Enterococcus* spp. izolowanych z moczu [Antibiotic susceptibility analysis of *Enterococcus* spp. isolated from urine]. PrzeglLek. (2004) 61:473–6. 15515808

[33] Yilema A Moges F Tadele S Endris M Kassu A Abebe W. Isolation of enterococci, their antimicrobial susceptibility patterns and associated factors among patients attending at the University of Gondar Teaching Hospital. BMC Infect Dis. (2017) 17:276. 10.1186/s12879-017-2363-3 28412932 PMC5392940

[34] Ramlakhan S Singh V Stone J Ramtahal A. Clinical options for the treatment of urinary tract infections in children. Clin Med Insights Pediatr. (2014) 24:8:31–7. 10.4137/CMPed.S8100 25210486 PMC4149380

[35] Kutasy B Coyle D Fossum M. Urinary tract infection in children: management in the era of antibiotic resistance-a pediatric urologist's view. Eur Urol Focus. (2017) 3:207–11. 10.1016/j.euf.2017.09.013 28965960

[36] Gupta K Hooton TM Naber KG Wullt B Colgan R Miller LG. International clinical practice guidelines for the treatment of acute uncomplicated cystitis and pyelonephritis in women: a 2010 update by the Infectious Diseases Society of America and the European Society for Microbiology and Infectious Diseases. Clin Infect Dis. (2011) 52:e103–e20. 10.1093/cid/ciq257 21292654

