# Supplementary material for: Evaluation of Antibiotic Prescribing Practices and Antimicrobial Sensitivity Patterns in Urinary Tract Related Infectious Diseases in Pediatric Patients
Source: Front Pediatr. 2021 Dec 23;9:740106. doi: 10.3389/fped.2021.740106 (PMC8734635; doi:10.3389/fped.2021.740106)
Supplement: Supplementary file 1 [file Table_1.DOCX]

| **Name of pathogens** | **Male (75)** | | **Female (57)** | | **Total (132)** | |
| --- | --- | --- | --- | --- | --- | --- |
|  | **Number** | **%** | **Number** | **%** | **Number** | **%** |
| Escherichia coli (E. coli) | 21 | 28 | 21 | 36.84 | 42 | 31.82 |
| Klebsiella pneumoniae | 21 | 28 | 12 | 21.05 | 33 | 25.00 |
| Pseudomonas aeruginosa | 6 | 8 | 3 | 5.26 | 9 | 6.82 |
| Enterococcus faecium | 9 | 12 | 0 | 0.00 | 9 | 6.82 |
| Klebsiella oxytoca | 3 | 4 | 3 | 5.26 | 6 | 4.55 |
| Mixed infections | 3 | 4 | 3 | 5.26 | 6 | 4.55 |
| Klebsiella pneumonia  and Pseudomonas aeruginosa | 3 | 4 | 0 | 0.00 | 3 | 2.27 |
| Klebsiella pneumoniae and E. coli | 0 | 0 | 3 | 5.26 | 3 | 2.27 |
| Staphylococcus aureus | 3 | 4 | 0 | 0.00 | 3 | 2.27 |
| Enterobacter colacae | 0 | 0 | 3 | 5.26 | 3 | 2.27 |
| Acinetobacter MDR | 3 | 4 | 0 | 0.00 | 3 | 2.27 |
| Staphylococcus epidermidis | 0 | 0 | 3 | 5.26 | 3 | 2.27 |
| Septicemia (Multiple infection) | 0 | 0 | 3 | 5.26 | 3 | 2.27 |
| Actinetobacter baumannii complex | 0 | 0 | 3 | 5.26 | 3 | 2.27 |
| Stenotrophomonas maltophilia | 3 | 4 | 0 | 0.00 | 3 | 2.27 |

**Supplementary data**

**Table 1: Distribution of uropathogens causing urinary tract infection**
